# Schmallenberg Virus in *Culicoides* spp. Biting Midges, the Netherlands, 2011

**DOI:** 10.3201/eid1901.121054

**Published:** 2013-01

**Authors:** Armin R.W. Elbers, Rudy Meiswinkel, Erik van Weezep, Marianne M. Sloet van Oldruitenborgh-Oosterbaan, Engbert A. Kooi

**Affiliations:** Central Veterinary Institute, Lelystad, the Netherlands (A.R.W. Elbers, E. van Weezep, E.A. Kooi);; Rocca di Cave, Rome, Italy (R. Meiswinkel);; and Utrecht University, Utrecht, the Netherlands (M.M. Sloet van Oldruitenborgh-Oosterbaan)

**Keywords:** Orthobunyaviridae, Schmallenberg virus, Culicoides obsoletus sensu stricto, Culicoides scoticus, Culicoides chiopterus, RT-PCR, viruses, biting midges, RNA, the Netherlands, SBV, Culicodes spp

## Abstract

To determine which species of *Culicoides* biting midges carry Schmallenberg virus (SBV), we assayed midges collected in the Netherlands during autumn 2011. SBV RNA was found in *C. scoticus*, *C. obsoletus sensu stricto*, and *C. chiopterus*. The high proportion of infected midges might explain the rapid spread of SBV throughout Europe.

During early summer 2011, Schmallenberg virus (SBV), a novel orthobunyavirus of the Simbu serogroup, spread across much of northern Europe, infecting ruminant livestock. The Simbu serogroup (family *Bunyaviridae*, genus *Bunyavirus*) includes Shamonda virus, Akabane virus, Sathuperi virus, and Aino virus. These viruses cause teratologic effects in ruminants and are arthropod-borne, and most have been isolated in the Old World from mosquitoes and *Culicoides* spp. biting midges (*1*). Recent preliminary studies indicate that ≥1 species of *Culicoides* midges act as field vectors for SBV in Europe (*2*). To determine which *Culicoides* midge species harbor SBV, we analyzed midges collected from 3 livestock holdings in eastern and northeastern parts of the Netherlands.

## The Study

Throughout September and early October 2011, *Culicoides* spp. biting midges were trapped almost daily at a dairy in the municipality of Ermelo (eastern Netherlands) by various methods, including the standard Onderstepoort-type blacklight trap. In addition, during several days in August and September 2011, *Culicoides* spp. biting midges were trapped near sheep flocks in the municipalities of Bilthoven (central Netherlands) and Midden-Drenthe (northeastern Netherlands) by using the Onderstepoort-type trap and a drop-tent cage. Captured midges were stored in 70% ethanol.

Female midges were categorized as nulliparous, parous, gravid, or freshly blood fed (engorged) (*3*); only midges belonging to the first 3 categories were assayed. The 6,100 selected midges were divided into 610 species-specific pools, 10 midges per pool. Under a dissecting microscope, the heads were separated from abdomens by use of a scalpel; 10 heads were then pooled and assayed for SBV, whereas the corresponding abdomens (also pooled) were stored in 70% ethanol.

All midges were identified morphologically, but because female *C. obsoletus* sensu stricto midges cannot be separated with confidence from *C. scoticus* midges, they were pooled and are referred to jointly as the *C. obsoletus* complex. The number of pools assayed for each species was as follows: *C. obsoletus* complex (230), *C. chiopterus* (144), *C. dewulfi* (130), *C. punctatus* (105), and *C. pulicaris* (1). After assays were conducted, the species identity of each SBV-positive midge pool was established by using molecular techniques.

Only when a pool of 10 heads was found SBV positive was the corresponding pool of dissected abdomens retrieved and assayed. In this instance, the 10 abdomens were assayed singly, so that the individual abdomen that was SBV-positive could be identified molecularly, to establish exactly which of the 2 species of the *C. obsoletus* complex was involved and to confirm or refute the morphologic identifications that had been made for the remaining *Culicoides* species.

RNA extraction was performed according to a protocol developed by CODA-CERVA (Centrum voor Onderzoek in Diergeneeskunde en Agrochemie, Centre d’Étude et de Recherches Vétérinaires et Agrochimiques), Brussels, Belgium; whereas, reverse transcription PCR (RT-PCR) was performed according to a method recently developed to detect the small segment of SBV (*4*). The RT-PCR cutoff value for the pooled heads was set at a cycle threshold (C_t_) value of 35. Pools with C_t_ >35 were retested and considered positive when confirmed. Reported C_t_ values for blood samples from infected cattle in Germany, tested by using the same RT-PCR, were 24–35 (*5*) and were used as the guide for our choice of cutoff value. If a specific pool of midge heads tested positive, individual abdomens from the corresponding stored pool were tested separately by RT-PCR. For molecular identification of the SBV-positive midges, the 18S internal transcribed spacer 1 (ITS1) 5.8S region was amplified by using the PanCulF and PanCulR primer set, adapted from Cêtre-Sossah et al. (*6*). The ITS1 sequences obtained from the SBV-positive abdomens were used to develop a *Culicoides* spp. phylogeny (Figure), which includes GenBank sequences representing all 5 species of the subgenus *Avaritia* (including *C. imicola*) known to be involved in the transmission of arboviruses in western Europe.

**Figure:**
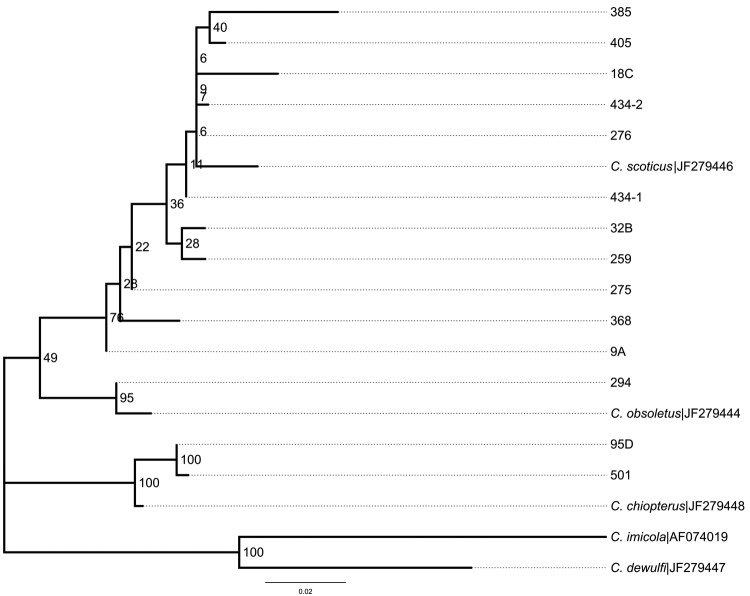
Phylogenetic tree comparing Schmallenberg virus–positive *Culicoides* spp. biting midge abdomens isolated in different regions in the Netherlands, 2011, with reference sequences from Deblauwe et al. (*7*). *C. imicola* was used as an outgroup. Bootstrap values are indicated at the significant nodes. Scale bar indicates nucleotide substitutions per site.

Of the 610 *Culicoides* midge head pools, 14 (2.3%) were SBV positive according to RT-PCR (Table 1): 11 *C. scoticus,* 1 *C. obsoletus* s.s., and 2 *C. chiopterus*. Of the 14 pools, 13 comprised midges from the dairy in Ermelo; midges in the remaining *C. chiopterus* pool came from Midden-Drenthe. C_t_ values for 12 of the 14 pools ranged from 19.6 to 30.44; C_t_ values for the remaining 2 pools were 34.98 and 36.78 (Table 2). C_t_ values for 13 of the individual midge abdomens linked to each pool of SBV-positive heads were lower (meaning a higher viral load) than those obtained for their corresponding heads. In 1 pool of *C. obsoletus* complex midges, 2 of 10 abdomens were positive for SBV, 1 strongly and 1 weakly. RT-PCRs for SBV were negative for all 130 pools of *C. dewulfi*, 105 pools of *C. punctatus*, and the 1 pool of *C. pulicaris* midges.

**Table 1:** Schmallenberg virus RNA in *Culicoides* spp. biting midges collected August–September 2011, the Netherlands

Municipality (Province)	Pools, no. positive/no. tested*					
	*C. obsoletus* complex	*C. dewulfi*	*C. chiopterus*	*C. punctatus*	*C. pulicaris*	Total
Bilthoven (Utrecht)	0/10	0	0	0	0	0/10
Midden-Drenthe (Drenthe)	0/5	0	1/39	0	0	1/44
Ermelo (Gelderland)	12/215	0/130	1/105	0/105	0/1	13/556
Total	12/230	0/130	2/144	0/105	0/1	14/610

*Tested by reverse transcription PCR.

**Table 2:** C_t_ values of Schmallenberg virus–positive *Culicoides* spp. biting midges collected August–September 2011, the Netherlands*

Pool no.	Pooled heads				Individual abdomens		
	Species identification by morphologic examination	C_t_ value			Species identification by DNA sequencing	C_t_ value	
		First test	Second test			First test	Second test
95-D	*C. chiopterus*	27.88	NA		*C. chiopterus*	24.59	NA
501	*C. chiopterus*	35.36	34.98		*C. chiopterus*	36.45	35.07
9-A	*C. obsoletus* complex	30.44	NA		*C. scoticus*	24.75	NA
18-C	*C. obsoletus* complex	28.24	NA		*C. scoticus*	24.95	NA
32-B	*C. obsoletus* complex	21.84	NA		*C. scoticus*	18.32	NA
259	*C. obsoletus* complex	19.60	NA		*C. scoticus*	18.16	NA
275	*C. obsoletus* complex	20.72	NA		*C. scoticus*	20.39	NA
276	*C. obsoletus* complex	36.02	36.78		*C. scoticus*	36.68	NA
293	*C. obsoletus* complex	20.43	NA		No reliable sequence	19.95	NA
294	*C. obsoletus* complex	24.60	NA		*C. obsoletus* sensu stricto	20.06	NA
368	*C. obsoletus* complex	25.21	NA		*C. scoticus*	21.80	NA
385	*C. obsoletus* complex	20.67	NA		*C. scoticus*	20.25	NA
405	*C. obsoletus* complex	23.38	NA		*C. scoticus*	21.64	NA
434†	*C. obsoletus* complex	23.68	NA		*C. scoticus*	23.10	NA

*C_t_ values determined by reverse transcription PCR. All midges were collected from cattle in the Ermelo municipality except no. 95-D, which was collected from sheep in the Midden-Drente municipality. C_t_, cycle threshold; NA, not applicable. †Two abdomens from this pool were positive; C_t_ values for the second abdomen were 35.75 and 35.37.

The species of all but 1 midge abdomen could be molecularly identified on the basis of ITS1 (Table 2). Not only did the molecular results confirm most of the morphologic identifications, but they also showed that *C. scoticus* seems to have played a more prominent role than *C. obsoletus* s.s. in transmission of SBV. The ITS1 sequences obtained from samples 95-D and 501 were almost identical to those published for *C. chiopterus*; the same applies to sample 294, which represented *C. obsoletus* s.s. (Table 2) (*7*). Although sequence polymorphism in *C. scoticus* was diverse, we were able to unambiguously assign each of the 11 SBV-positive abdomens to this species (Figure).

Prevalence of SBV among the *Culicoides* spp. midges was 0.25% (15/6,100 midges tested). More specifically, the prevalence of SBV in the 2 species that comprised the *C. obsoletus* complex was 0.56% (13/2,300 tested). This prevalence is similar to that obtained for Akabane virus in *C. brevitarsis* midges from Australia (*8*–*11*) but about 10× higher than that reported for bluetongue virus (*12*). For *C. chiopterus* midges, prevalence of SBV was 0.14% (2/1,440 tested), ≈5× higher than prevalence of bluetongue virus (*13*).

## Conclusions

Our results demonstrate that SBV was harbored in 3 species of field-collected *Culicoides* biting midges: *C. scoticus, C. chiopterus*, and *C. obsoletus* s.s. These species were among the more abundant of the 15 species found at the livestock holdings sampled. The holdings were situated in the center of the epidemic area, and of the ≈100 animals at the dairy in Ermelo, >96% had seroconverted to SBV. The low C_t_ values indicate that concentrations of the virus in most SBV-positive *Culicoides* midges were high. The fact that the C_t_ values for the heads of midges matched closely with those from the associated abdomens renders it certain that SBV had replicated to transmissible levels in these midges and supports the contention that 2 species of the *C. obsoletus* complex, along with *C. chiopterus,* act as natural vectors for SBV. Despite the relatively large numbers of SBV-negative pools, our findings should not be interpreted to exclude the involvement of other species, such as *C. dewulfi* or *C. punctatus*, in field transmission of SBV. We conclude that the high proportion of SBV-positive *Culicoides* spp. midges and the multiple vector species could help explain the rapid spread of SBV throughout much of Europe during 2011.
